# Case report: Pathological complete response to perioperative treatment of radiotherapy combined with angiogenesis inhibitor in a patient with pleomorphic liposarcoma

**DOI:** 10.3389/fonc.2023.925233

**Published:** 2023-01-27

**Authors:** Chenlu Zhang, Wenshuai Liu, Binliang Wang, Na Zhu, Xi Guo, Zhiming Wang, Rongyuan Zhuang, Yang You, Yong Zhang, Hanxing Tong, Weiqi Lu, Yuhong Zhou

**Affiliations:** ^1^Department of Medical Oncology, Zhongshan Hospital, Fudan University, Shanghai, China; ^2^Department of General Surgery, Zhongshan Hospital, Fudan University, Shanghai, China; ^3^Department of Radiation, Zhongshan Hospital, Fudan University, Shanghai, China; ^4^Department of Pathology, Zhongshan Hospital, Fudan University, Shanghai, China

**Keywords:** pleomorphic liposarcoma, preoperative radiotherapy, angiogenesis inhibitor, pathological complete response, case report

## Abstract

**Background:**

Liposarcomas (LPS) are mesenchymal malignancies with four principal subtypes presenting distinct molecular and clinical features. Pleomorphic liposarcoma (PLPS) is one of the rarest and most aggressive subtypes of LPS. Surgical resection is currently a preferred curative approach for localized PLPS. However, the prognosis of unresectable PLPS is extremely poor, and there is no standard treatment.

**Case presentation:**

A 59-year-old Chinese woman was diagnosed with unresectable PLPS. The case was discussed and managed by specialists from a multidisciplinary team at Fudan Zhongshan Hospital. Preoperative radiotherapy (RT) of intensity-modulated radiation therapy (IMRT) at 50 Gy/25 Fx concurrently with the angiogenesis inhibitor anlotinib (8 mg, days 1–14, every 3 weeks) was prescribed to the patient. The dosage of anlotinib was increased to 10 mg after RT. After 6 months of treatment, the tumor had significantly shrunk and was successfully resected. Examination of the surgical specimens showed a pathological complete response (pCR). Until the latest follow-up (April 2022), no recurrence was observed, and disease-free survival has exceeded 14 months.

**Conclusion:**

This case sheds light on the probability that perioperative RT combined with an angiogenesis inhibitor can be effectively used in PLPS, which is resistant to chemotherapy and usually considered to have a poor prognosis. Further studies with randomized controlled clinical trials will improve our knowledge of this preoperative treatment strategy.

## Introduction

Liposarcoma (LPS) is a heterogeneous soft tissue sarcoma. LPS is among the most common soft tissue sarcomas (STS) and accounts for approximately 15% to 20% of all STS (1). Pleomorphic liposarcoma (PLPS), a less frequent but more aggressive subtype with a 5-year survival rate of 57%, which is closer to that of other high-grade STS, accounts for only 5%–10% of LPS (2).

The LPS arising in the retroperitoneum and intra-abdomen is recommended to be evaluated and managed by a multidisciplinary team (MDT) according to the latest NCCN guideline. Surgery is currently the mainstay, but patients with unresectable disease—defined as tumors affecting important structures or causing unacceptable morbidity after excision—should consider perioperative treatment. Perioperative radiotherapy (RT) is one option, as it reduces tumor size and facilitates tumor resection (3). However, evidence regarding perioperative RT is limited. To date, the STRASS trial (NCT01344018) is the first randomized, phase III clinical trial to value the role of preoperative RT for localized retroperitoneal STS (RPS) (4). Unfortunately, this trial showed a negative result in that preoperative RT did not improve recurrence-free survival. A subgroup analysis of patients with LPS suggested a 10% increase in recurrence-free survival in the RT plus surgery group.

Angiogenesis inhibitors such as anlotinib have recently been proven to be effective in advanced and metastatic STS (5). Although angiogenesis inhibitors are not recommended as perioperative treatment in the guidelines, some recent early-phase clinical trials have explored the safety and efficacy of angiogenesis inhibitors in STS, mostly in combination with perioperative RT (6). These trials showed that the combination of antiangiogenesis therapy with perioperative RT is worth trying in individually selected patients.

Owing to the low incidence of PLPS and the lack of related investigations, the optimal perioperative treatment regimen is challenging. Here, we report a case of PLPS with a pathological complete response (pCR) after RT combined with an antiangiogenesis drug as perioperative therapy.

## Case description

**Figure 1:**
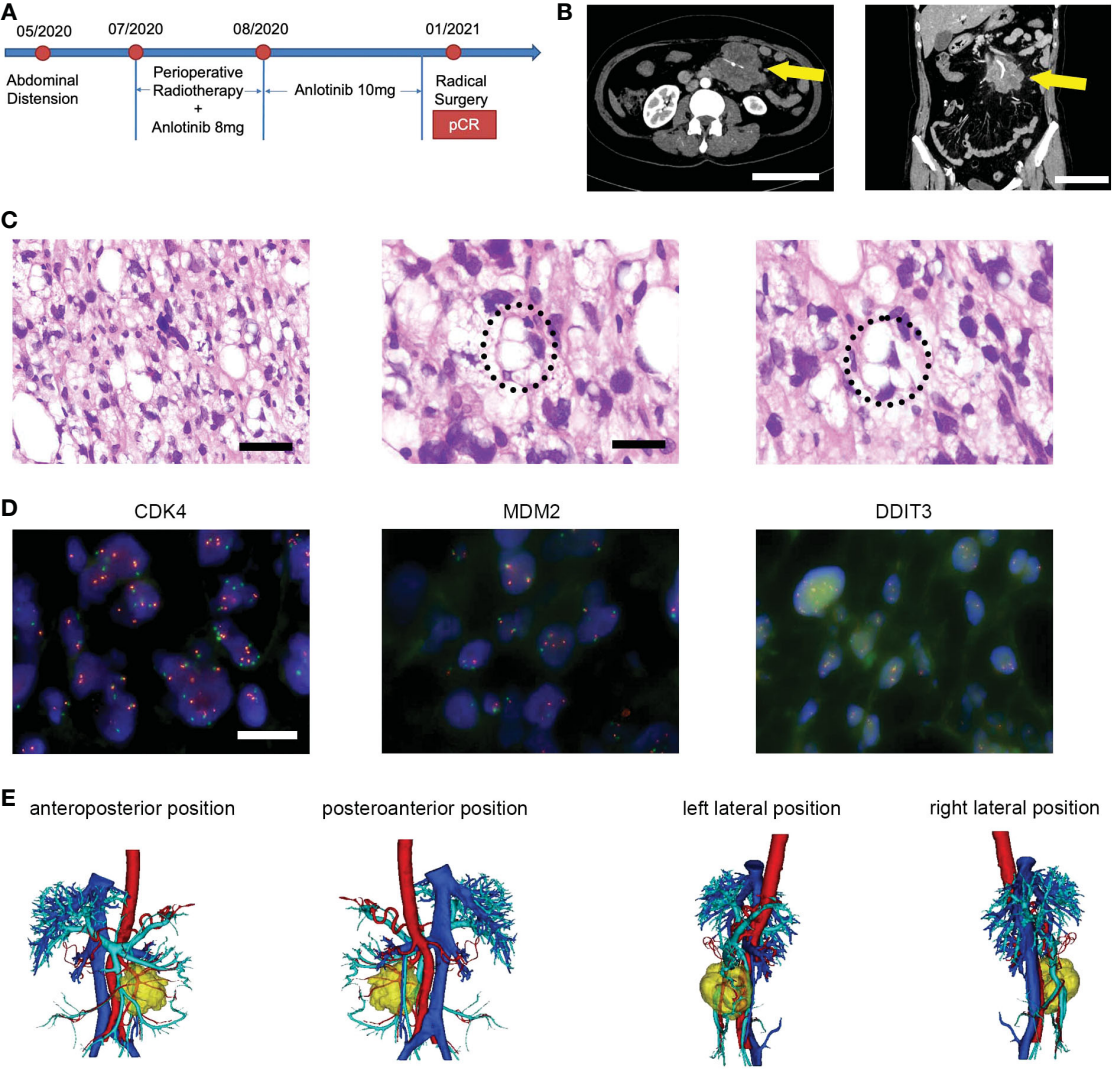
**(A)** Timeline of therapy in a patient with unresectable PLPS who received preoperative RT plus anlotinib and achieved pCR after the surgery. **(B)** The initial CT scan revealed a soft tissue mass in the left abdominal cavity with the superior mesenteric artery passing through, measuring 7.4 cm × 6.2 cm × 7.2 cm in diameter. Scale bar = 10 cm. **(C)** Micrographs of hematoxylin and eosin (HE) staining. Scale bar = 50 μm. The latter two images showed the lip blasts in a circle. Scale bar = 25 μm. **(D)** The FISH image showed negative results labeled by CDK4, MDM3, and DDIT3 probes, respectively. Scale bar = 10 μm. **(E)** 3D reconstruction imaging displayed the relationship between tumors and vessels. Yellow, tumor; red, artery; blue, vein.

**Figure 2:**
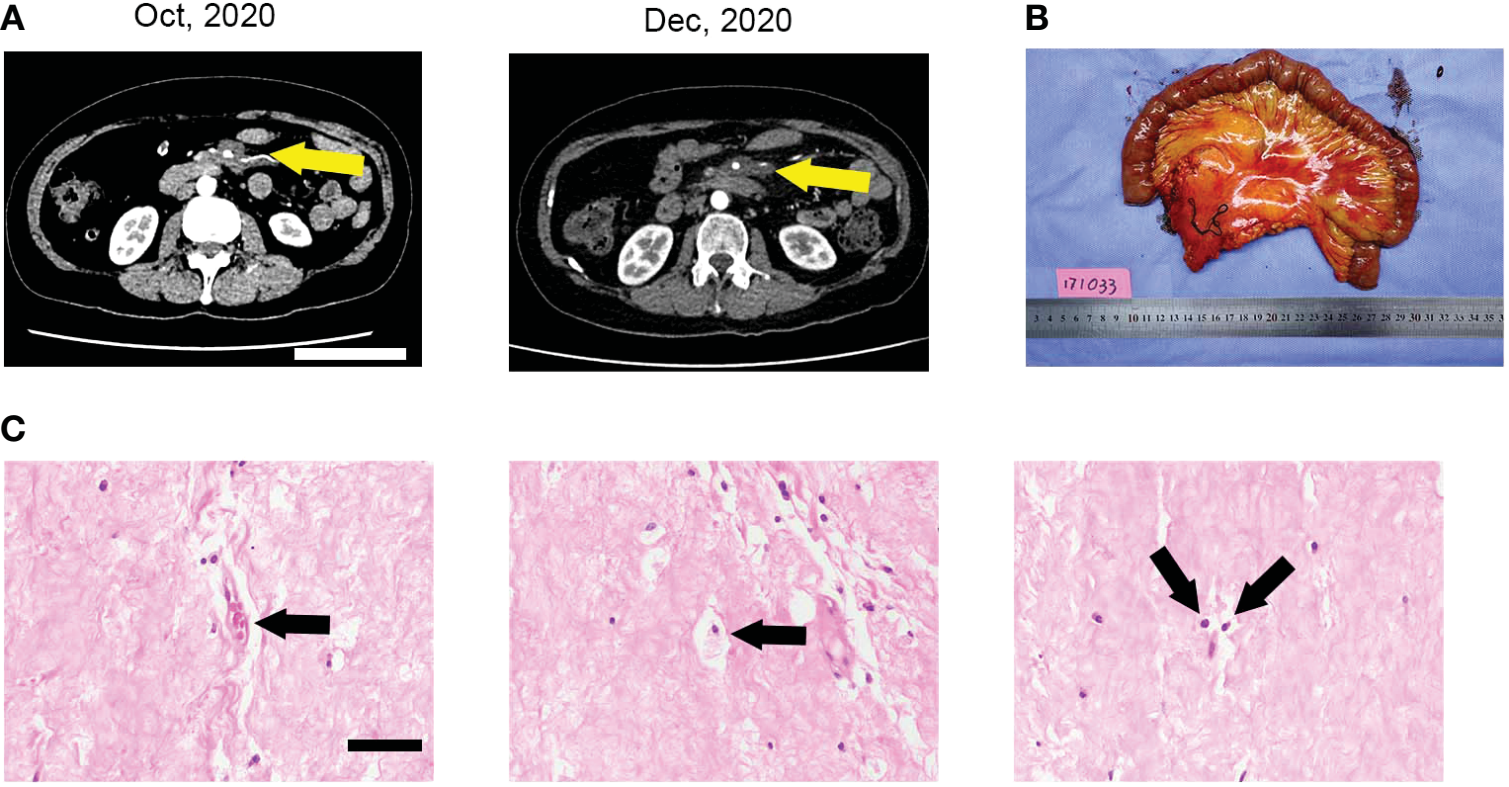
**(A)** The CT scan showed that the tumor shrank remarkably after the treatment of perioperative RT plus anlotinib, measuring 2.8 cm × 1.9 cm. **(B)** The surgical specimens of the tumor, partial small intestines, mesentery, and superior mesenteric artery. **(C)** Micrographs of HE staining showed collagen tissue with no viable tumor cells. The images displayed blood vessels, histiocytes, and chronic inflammatory cells, which were pointed with arrowheads.

The case management is described below, and **Figure 1A** provides a detailed timeline. A 59-year-old Chinese woman presented with abdominal distension. The results of the blood test were normal, and the patient did not have a family history of malignancies. The performance status measured by the ECOG score was one. Computed tomography (CT) was performed in June 2020 and showed a soft tissue mass, sized 7.4 cm × 6.2 cm ×7.2 cm, in the left abdominal cavity with the superior mesenteric artery passing through (**Figure 1B**). A biopsy was performed, and histopathology revealed undifferentiated pleomorphic epithelioid or spindled cells, admixed with some cells presenting lipid vacuoles (**Figure 1C**). Lipoblasts could be found in the biopsy sections. The immunohistochemistry reading was ERG (−), CD34 (vessels+), CD117 (−), Fli-1 (−), OCT4 (−), SMA (−), Ki67 (20%+), CD31 (−), DOG1 (−), S100 (−), Des (−), and MDM2 (100%++). Fluorescence *in situ* hybridization (FISH) revealed no amplification of MDM2 and CDK4 and no rearrangement of DDIT3 (**Figure 1D**). The diagnosis was pleomorphic liposarcoma, staging cT2N0M0G2, IIIA based on the above information and pathological evaluation of the biopsy. Furthermore, 3D reconstruction imaging confirmed that the first branch of the superior mesenteric artery was surrounded by the tumor (**Figure 1E**). The careful preoperative imaging helped the MDT to comprehensively evaluate the case. At that time, immediate surgery would necessitate a resection of the entire small bowel to completely remove the tumor, and the patient would need lifelong parenteral nutrition afterward. Consequently, preoperative RT combined with an antiangiogenesis drug was suggested, and the patient agreed with it. From July 2020 to August 2020, the patient received intensity-modulated radiation therapy (IMRT) of 50 Gy/25 Fx concurrently with anlotinib (8 mg, days 1–14, every 3 weeks). The anlotinib dosage was increased to 10 mg after RT. The therapy was well tolerated, and the main adverse event was G1 fatigue. Follow-up CT scans were performed in October and December 2020, indicating notable tumor shrinkage to 2.8 cm × 1.9 cm (**Figure 2A**). On 29 January 2021, the patient underwent radical resection of the lesion and partial resection of the superior mesenteric artery and small intestines (**Figure 2B**). The surgical margins showed no evidence of tumor involvement. The pathology of surgical specimens displayed adipose tissue composed of large areas of hyperplastic collagen tissue, scattered small blood vessels, histocytes, and chronic inflammatory cells (**Figure 2C**). The diagnosis results revealed a pCR. After surgery, the patient underwent CT and blood tests every 3 months. At the last follow-up in April 2022, 14 months postsurgery, the patient was in good condition, and no suspicious recurrence was detected.

## Discussion

PLPS is the rarest and most aggressive subtype of LPS, and there is currently no standardized treatment yet. Complete resection with clear surgical margins is the preferred curative option for localized PLPS (7). However, local recurrence of PLPS is approximately 30%–50% (8), which is the reason for the predominant failure of surgical treatment. Perioperative treatment may resolve this issue by reducing local recurrence. In this case, although the follow-up of 14 months was rather short, the patient did not develop a local or distant recurrence during this time.

The results of a systematic review and meta-analysis indicated that external beam radiation therapy could reduce local recurrence in RPS (odds ratio (OR) = 0.47, *p <*0.0001), which was less frequent in the preoperative RT group than the postoperative group (OR = 0.03, *p* = 0.02) (9). Another study showed that patients with intermediate or high-grade RPS could benefit from the treatment with preoperative RT plus complete resection, with 5-year local recurrence-free survival (RFS) of 60%, disease-free survival (DFS) of 46%, and overall survival (OS) rates of 61% (10). Additionally, retrospective studies have revealed that using perioperative RT in combination with surgery for RPS could also improve OS. The analysis showed that the median OS was significantly improved in the perioperative RT plus surgery group compared to the surgery-only group, which were 110 and 66 months, respectively (hazard ratio = 0·70, 95% confidence interval = 0·59–0·82; *p* < 0.0001) (11).

However, in contrast to extremity STS (3, 12), the role of preoperative RT in RPS still lacks relevant high-grade clinical evidence. The STRASS trial is a prominent randomized clinical trial that aimed to evaluate the effect of preoperative RT on RPS (4). There was no improvement in abdominal recurrence-free survival (ARFS) in patients receiving preoperative RT plus surgery compared to surgery alone. This study was hobbled by several key limitations, as ARFS was a complex primary endpoint and the trial was not histotype-specific. Nevertheless, the STRASS trial provided some suggestive evidence regarding the possible benefit of preoperative RT in specific RPS histologic subtypes, including LPS and low-grade sarcoma subgroups. Further histotype-specific investigations are still required.

Conventional chemotherapy shows a low response in PLPS; therefore, new target therapies are urgently needed. Vascular endothelial growth factor (VEGF) expression was observed in 68% of PLS specimens, which is an excellent sign of the potential benefit of angiogenesis inhibitors (13). Angiogenesis inhibitors, including pazopanib, anlotinib, and regorafenib, have been approved for use in advanced and metastatic STS (5, 14, 15). Anlotinib is a multi-targeted tyrosine kinase inhibitor that selectively targets VEGFR-2, VEGFR-3, and VEGFR-4; FGFR-1, FGFR-2, FGFR-3, and FGFR-4; PDGFR; and c-Kit, contributing to reduced tumor growth and vasculature (16). A phase II clinical trial showed that anlotinib was the first TKI with antitumor activity in LPS patients that progressed after standard first-line therapy. The progression-free rate (PFR)_12 weeks_ and the objective response rate (ORR) was 63% and 7.7% in LPS (17). However, there is no clear recommendation for locally advanced PLPS and whether angiogenesis inhibitors can be used in perioperative treatment.

A combination of RT and angiogenesis inhibitors has been demonstrated to be an effective therapy that enhances the sensitivity of tumor cells to radiation (6, 18). This effect can be explained by the fact that anti-VEGF normalizes tumor vasculature, resulting in increased tumor oxygenation and cytotoxicity to radiation (19). Combination treatment has been proven to increase the efficacy of RT in different malignancies. Several trials have evaluated the addition of bevacizumab, a humanized anti-VEGF monoclonal antibody, in the treatment of rectal cancer along with preoperative chemoradiotherapy (20–22). Higher pCR rates were observed in the bevacizumab group, ranging from 23.8% to 39.5%. Early clinical studies have also been conducted on STS. Sunitinib or bevacizumab was administered concurrently with preoperative RT in patients with STS originating from the extremities, retroperitoneum, and trunk (23–25). Remarkably, the pathological examination of tumor specimens revealed less than 10% viable tumor cells in approximately one-third of the patients after the combination treatment. Moreover, combination treatment did not lead to severe adverse events or affect the dose of RT. This implied that the addition of angiogenesis inhibitors to RT could increase RT efficacy without increasing toxicity.

In summary, to the best of our knowledge, this is the first case report of a pathologically complete response to the combination of perioperative RT and antiangiogenic agents in a patient with primary unresectable PLPS. Combined therapy provides a practical therapeutic approach to overcome the obstacles in PLPS that have no standard treatment. Further studies will help us better understand the underlying molecular mechanisms of PLPS and establish an optimal treatment strategy.

## Data availability statement

The original contributions presented in the study are included in the article/Supplementary Material. Further inquiries can be directed to the corresponding authors.

## Ethics statement

The studies involving human participants were reviewed and approved by Ethics Committee of Zhongshan Hospital, Fudan University. The patients/participants provided their written informed consent to participate in this study. Written informed consent was obtained from the individual(s) for the publication of any potentially identifiable images or data included in this article.

## Author contributions

CZ and WSL collected the clinical information, diagnostic information, therapeutic information, and images of the patients. BW provided the radiotherapy information. YZ and HT provided the surgical information and images. NZ reviewed the pathological sections and took pathological photos. CZ wrote the manuscript. CZ and WSL revised the manuscript. YHZ proofread the manuscript. YHZ and WQL were responsible for the study’s conception and design. XG, ZW, RZ, and YY took part in the management and follow-up of the patient. All authors contributed to the article and approved the submitted version.
